# Near-Fatal Gastrointestinal Hemorrhage in a Child with Medulloblastoma on High Dose Dexamethasone

**DOI:** 10.7759/cureus.1442

**Published:** 2017-07-07

**Authors:** Derek Yecies, Daniel Tawfik, Jennifer Damman, Chad Thorson, David S Hong, Gerald A Grant, Rachel Bensen, Mihaela Damian

**Affiliations:** 1 Department of Neurosurgery, Stanford University School of Medicine; 2 Division of Pediatric Critical Care Medicine, Department of Pediatrics, Stanford University School of Medicine and Lucile Packard Children’s Hospital; 3 Division of Gastroenterology, Hepatology and Nutrition, Department of Pediatrics, Stanford University School of Medicine and Lucile Packard Children’s Hospital; 4 Division of Pediatric Surgery, Department of Surgery, Stanford University Stanford University School of Medicine and Lucile Packard Children’s Hospital; 5 Division of Pediatric Neurosurgery, Department of Neurosurgery, Stanford University School of Medicine and Lucile Packard Children’s Hospital; 6 Division of Pediatric Critical Care, Department of Pediatrics, Stanford University School of Medicine and Lucile Packard Children’s Hospital

**Keywords:** gastrointestinal hemorrhage, ulcer, cushing's ulcer, steroids, pediatric, medulloblastoma

## Abstract

A four-year-old female was admitted to a university-based children's hospital with a newly-diagnosed posterior fossa tumor. She was started on famotidine and high-dose dexamethasone and underwent gross total resection of a medulloblastoma. She was continued on dexamethasone and famotidine. She exhibited postoperative posterior fossa syndrome and was started on enteral feeds via the nasoduodenal tube. She had small gastrointestinal bleeds on postoperative days eight, 11, and 18, and was found to have a well-circumscribed posterior duodenal ulcer. On postoperative day 19, she suffered a massive life-threatening gastrointestinal bleed requiring aggressive resuscitation with blood products. She required an emergent laparotomy due to ongoing blood loss and she was found to have posterior duodenal wall erosion into her gastroduodenal artery. She recovered and subsequently began delayed chemotherapy. This case demonstrates a rare and life-threatening complication of high-dose dexamethasone therapy in the setting of posterior fossa pathology despite stress ulcer prophylaxis. We present a historical perspective with the review of the association between duodenal and intracranial pathology and the usage of high-dose dexamethasone in such cases.

## Introduction

In 1932, Dr. Harvey Cushing reported a series of patients found to have gastroduodenal ulcers after the initial presentation for brain tumors [1]. He suggested possible pathophysiologic mechanisms responsible for the observed link between space-occupying lesions in the brain and gastrointestinal ulceration. All three children in the case series died of neurosurgical or operative complications and were found to have gastrointestinal ulcerations on postmortem examination.

Gastroduodenal ulceration secondary to intracranial pathology has now been termed a Cushing’s ulcer, but reports of severe sequelae in the pediatric population remain infrequent, attributed to the advent of acid suppression [2]. We present a child with life-threatening duodenal ulceration after successful resection of a posterior fossa tumor, despite acid suppression. Informed consent statement was obtained for this study.

## Case presentation

A four-year-old female with a history of prematurity (31 weeks gestation), atrial septal defect, and neonatal tachycardia requiring digoxin for the first year of life was admitted to a university-based children’s hospital following evaluation in an emergency department for one month of progressive ataxia, several days of headache, and two days of emesis. Brain magnetic resonance imaging (MRI) at the outside hospital revealed ventriculomegaly and a posterior fossa mass. She initially received 7.5 mg (0.5 mg/kg) dexamethasone, followed by 4 mg dexamethasone every six hours (one mg/kg/day) and standard–dose IV famotidine 0.25 mg/kg BID for the first three days of admission. On hospital day two, she underwent intraoperative external ventricular drain (EVD) placement and a suboccipital craniotomy with gross total resection of her tumor which was ultimately found to be a grade four, C-myc amplified (group three) medulloblastoma, initially staged M0. She received 6 mg dexamethasone at the beginning of the case and the tumor was noted to be invasive into the floor of the fourth ventricle and the bilateral middle cerebellar peduncles. Postoperative magnetic resonance imaging (MRI) demonstrated no residual tumor or stroke, though there was some indeterminate enhancement in the infundibular recess of the third ventricle. Her immediate postoperative course was notable for mutism and lethargy, which would continue as a severe case of transient posterior fossa syndrome. She was continued on dexamethasone 2 mg every six hours, which was lowered to 1 mg every six hours on the postoperative day five with twice daily ranitidine (2 mg/kg/dose) continued throughout. Enteral feeds were initiated on postoperative day one via the nasoduodenal tube.

**Figure 1:**
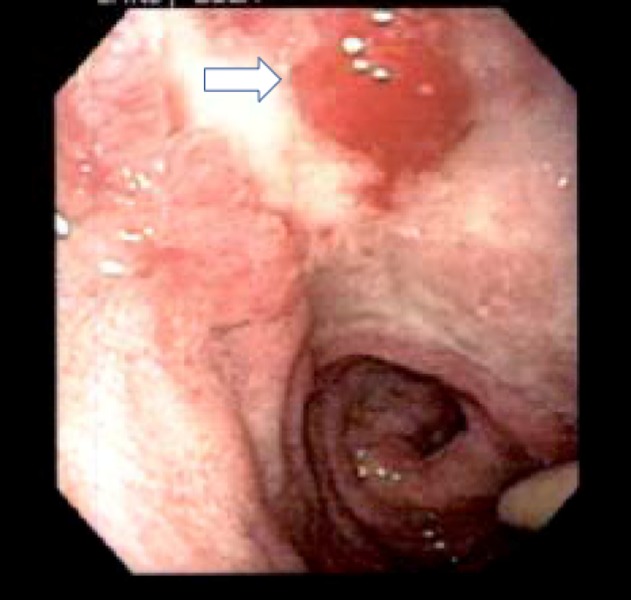
Endoscopic view of posterior duodenal ulcer (indicated by white arrow) in the patient

On the postoperative day eight, she passed bright red blood per rectum with sudden onset of tachycardia to over 220 beats per minute and anemia with hemoglobin 4.3 g/dL. She stabilized with red blood cell transfusions, pantoprazole infusion, and initiating a rapid two-day dexamethasone wean. Despite treatment with a proton pump inhibitor (PPI), she had repeat hematochezia on postoperative day 11, which required additional red blood cell transfusion. Esophagogastroduodenoscopy showed a large, well-circumscribed posterior duodenal ulcer with no active bleeding (Figure 1), and no therapeutic intervention was delivered. An enteric tube tip found to be near this lesion was withdrawn to terminate in the stomach. Her external ventricular drain (EVD) was removed on postoperative day 13 and she was transferred to the pediatric ward.

She was gradually advanced to feeds via nasogastric tube and transitioned to twice daily standard dosing of IV pantoprazole by postoperative day 16 without any clinical signs of bleeding until postoperative day 18 when the patient was noted to be tachycardic with an acute decrease in hemoglobin from 9.4 to 7.2 g/dL but no clinical evidence of bleeding, prompting return to the intensive care unit for closer observation. She was placed on a pantoprazole infusion for one day, then resumed nasogastric PPI and feeds when hemoglobin (Hgb) remained stable. On postoperative day 19, massive gastrointestinal bleed and hemorrhagic shock were signaled by sudden tachycardia to 210, progression to diffuse pallor, poor responsiveness, and hypotension within several seconds, with estimated blood loss of 1.8 liters per rectum was found. Her airway was secured and she required emergent resuscitation with three liters of blood products and vasoactive infusions. Upon emergent laparotomy, the stomach and duodenum were filled with arterial blood. Duodenotomy revealed active pulsatile bleeding from her gastroduodenal artery due to erosion from a posterior duodenal ulcer. Hemostasis was achieved by ligating the gastroduodenal artery and overseeing the duodenal ulcer. Due to diffuse bowel edema secondary to massive fluid resuscitation, her abdomen was left open with a temporary abdominal silastic dressing. Over the next four days, she underwent abdominal wall silo closure until the fascia was able to be closed.

Individualized chemotherapy as per clinical study ID SJYC07 was started on postoperative day 38, and she was found to have probable metastasis in the infundibular recess of the third ventricle on repeat brain MRI. The remainder of her postoperative course was complicated by Enterobacter cloacae bacteremia (E. cloacae) (presumed from intestinal translocation) requiring removal of her port and further delay in chemotherapy. Her posterior fossa syndrome was largely resolved at her three-month postoperative follow-up and she is now talking and walking with no focal neurologic deficit and MRI at one year demonstrated no residual or recurrent tumor. She was continued on pantoprazole for the first six months and is now 12 months postoperative with no further evidence of bleeding.

## Discussion

Although neurogenic gastroduodenal ulcers are often eponymously associated with Cushing, recognition of a connection between central nervous system disease and gastrointestinal disease extends as far back as 1772, when John Hunter published postmortem findings of gastromalacia in the patients who suffered fatal head trauma [3]. Rokitansky and Schiff further supported Hunters findings, with Rokitansky suggesting gastric hyperacidity as an etiology [4], further confirmed by subsequent studies [5-6]. Cushing presented several possible explanations for this connection, one of which implicated increased vagal tone. Expanding on the work done by Rokitansky and Mogilnitzkie [7], he outlined a theory of “parasympathetic stimulation within, or possibly from vagal release due to sympathetic paralysis” as the source of gastric hyperacidity and subsequent ulceration [1], a theory which remains largely unchanged to this day.

In the era prior to the routine administration of gastrointestinal prophylaxis with H2 blockers, the incidence of massive GI hemorrhage in pediatric patients with brain tumors was reported more commonly than expected. Lewis reported 10 cases of massive GI hemorrhage secondary to duodenal ulcers in approximately 100 pediatric patients with brain tumors during a five-year period [8]. Similarly, Ross reported three massive GI hemorrhages in 29 children with posterior fossa tumors during a one-year period [9]. Lewis reported the highest incidence of GI hemorrhage in the first seven to 10 postoperative days after craniotomy and noted that all of the children who suffered GI hemorrhages also displayed ‘acute and severe embarrassment of the brain-stem function at some point [8]. Although no single intracranial source has been implicated, the majority of the described cases are in patients with tumors of the posterior fossa, with the remainder being in the brainstem, thalamus, or hypothalamus [9]. Management of massive GI hemorrhage in these series was with vagotomy and pyloroplasty. Notably, Ross reported an elimination of massive GI hemorrhages in the year following the institution of the routine H2 blockade as prophylaxis in this population [9].

Our case is similar to those presented by others in that it involves posterior duodenal bulb ulceration in the setting of a posterior fossa tumor with brainstem invasion. The patient’s immediate onset of mutism and lethargy postoperatively is consistent with Lewis’ description of ‘embarrassment of brain-stem function.’ Ross presents a strikingly similar four-year-old with medulloblastoma who suffered hemorrhagic shock from erosion of an ulcer into his gastroduodenal artery following craniotomy. However, our case differs in two key ways. First, our patient received ulcer prophylaxis with appropriately-dosed H2 blocker and proton pump inhibitors (PPI) per standard of care in keeping with the lessons learned from prior similar cases, but this was insufficient to prevent massive gastrointestinal bleed. Second, although she evidenced smaller gastrointestinal bleeds in the common seven to 10-day postoperative time period, her massive life-threatening hemorrhage did not occur until postoperative day 19. If she had not had her early bleeding episodes or if they went under-recognized, she would have been home or in the acute care ward at the time of her massive hemorrhage. In either case, it is quite unlikely that her resuscitation could have been rapid enough to preserve her life, similar to a recently reported case where a 10-year-old male who suffered massive GI bleeding in the clinic and subsequently died despite ranitidine prophylaxis [10]. The administration of high-dose dexamethasone (recommended dosing 0.25-1 mg/kg/day) on presentation is standard for patients with posterior fossa tumors, however, our patient’s dosing at 1 mg/kg/day was at the high end of recommended dosing in children. Given the known dose-dependent effects of dexamethasone on increasing gastric acid secretion and inhibiting local repair mechanisms, this also may have been a contributing factor.

## Conclusions

This case supports the association between posterior fossa tumors and posterior duodenal ulceration but suggests that the current standard of acid suppression may not be sufficient to prevent life-threatening sequelae. Our experience emphasizes the need for prolonged vigilant observation, particularly in patients with known ulcerations, as continued ulceration may result in quite delayed hemorrhagic events. Given the preponderance of duodenal ulcers in this population, we suggest avoiding nasoduodenal feeding tubes to avoid any contribution of local inflammation from the feeding tube tip. Additionally, gastric pH monitoring may help guide acid suppressive therapy, with possible early dual prophylaxis with proton pump inhibitors (PPI) and H2 blocker. While we recognize the paucity of data on the ideal dosing of dexamethasone in this population, strong consideration of the increased risk of gastrointestinal (GI) bleeding associated with higher doses of dexamethasone should be given. Early endoscopy can be useful in localizing bleeding and for potential intervention. However, given the limitations of therapeutic endoscopy in small children along with its associated risks, aggressive early surgical management of hemorrhagic ulcers in children with posterior fossa tumors may be warranted.
